# Correlations between three ELISA protocols measurements of RTS,S/AS01-induced anti-CSP IgG antibodies

**DOI:** 10.1371/journal.pone.0286117

**Published:** 2023-05-23

**Authors:** Robert M. Mugo, Benedict Orindi, Faiz M. Shee, Duncan Bellamy, Jedidah Mwacharo, Katie J. Ewer, Philip Bejon, Francis M. Ndungu

**Affiliations:** 1 KEMRI-Wellcome Trust Research Programme, Kilifi, Kenya; 2 Department of Biological Sciences, Pwani University, Kilifi, Kenya; 3 Center for Infection Medicine, Institute of Immunology, Freie Universtät Berlin, Berlin, Germany; 4 Nuffield Department of Medicine, The Jenner Institute, University of Oxford, Oxford, United Kingdom; 5 Division of Infectious Diseases, Department of Medicine Solna and Centre for Molecular Medicine, Karolinska Institute, Stockholm, Sweden; Fundação Oswaldo Cruz Centro de Pesquisas René Rachou: Fundacao Oswaldo Cruz Instituto Rene Rachou, BRAZIL

## Abstract

**Background:**

RTS,S/AS01 induced anti-circumsporozoite protein (CSP) IgG antibodies are associated with the vaccine efficacy. There is currently no international standardisation of the assays used in the measurement of anti-CSP IgG antibody concentrations for use in evaluations of the vaccine’s immunogenicity and/or efficacy. Here, we compared the levels of RTS,S/AS01 induced anti-CSP IgG antibodies measured using three different enzyme-Linked ImmunoSorbent Assays (ELISA).

**Methods:**

196 plasma samples were randomly selected from the 447 samples collected during the RTS,S/AS01 phase IIb trial in 2007 from Kenyan children aged between 5–17 months. The vaccine-induced anti-CSP IgG antibodies were then measured using two independently developed ELISA protocols (‘Kilifi-RTS,S’ and ‘Oxford-R21’) and compared to the results from the reference ‘Ghent-RTS,S’ protocol for the same participants. For each pair of protocols, a deming regression model was fitted. Linear equations were then derived to aid in conversions into equivalent ELISA units. The agreement was assessed using Bland and Altman method.

**Findings:**

The anti-CSP IgG antibodies measured from the three ELISA protocols were in agreement, and were positively and linearly correlated; ‘Oxford’ and ‘Kilifi’ r = 0.93 (95% CI 0.91–0.95), ‘Oxford’ and ‘Ghent’ r = 0.94 (95% CI: 0.92–0.96), and ‘Kilifi’ and ‘Ghent’ r = 0.97 (95% CI: 0.96–0.98), p<0.0001 for all correlations.

**Conclusions:**

With the linearity, agreement and correlations established between the assays, conversion equations can be applied to convert results into equivalent units, enabling comparisons of immunogenicities across different vaccines of the same CSP antigens. This study highlights the need for the international harmonisation of anti-CSP antibody measurements.

## Introduction

Malaria is a major public health problem worldwide causing approximately 247 million cases and 619,000 deaths in 2021 [1]. Adoption of control strategies such as sleeping under insecticide-treated nets, and indoor residual insecticide spraying has contributed to a gradual reduction of malaria cases over the years. However, since 2015 the overall reduction in malaria incidence rate has stalled, with the mortality rate increasing slightly in 2020 [1]. Thus, there remains a need to develop and adopt novel control strategies such as the development of malaria vaccines.

RTS,S/AS01 malaria vaccine has been recommended and prequalified by the World Health Organization (WHO) for use in children at risk of *P*. *falciparum* malaria infections [1, 2]. The vaccine is expected to give new energy to the stalled fight against malaria [1]. RTS,S/AS01 is a sub-unit pre-erythrocytic malaria vaccine based on the *P*. *falciparum* circumsporozoite protein (CSP), fused to hepatitis B surface antigen (HBsAg). CSP is the immunodominant surface protein in the sporozoites membrane, containing known B and T-cells epitopes. CSP contains a central NANP repeat region and conserved flanking domains—a 4-amino acid sequence at the N-terminus and a type I Thrombospondin Repeat motif at the C-terminus of the repeats [3].

RTS,S/AS01 reported an efficacy of 46% from the large phase III clinical trials, 18 months following the third vaccine dose [4]. Further modification of RTS,S/AS01 may aid in boosting this efficacy and/or help in meeting the anticipated high demand for malaria vaccines. R21, a similar pre-erythrocytic malaria vaccine also based on CSP is currently undergoing Phase I and II clinical trials [5]. In contrast to RTS,S/AS01 which comprises 20% CSP, with the rest 80% being HBsAg, R21 comprises only fusion CSP protein moieties thus giving a greater coverage of the CSP aimed at improving the vaccine immunogenicity and subsequent efficacy [6]. Administration of R21 in combination with the Matrix-M^™^ (MM) adjuvant is safe and exhibits promising vaccine efficacy of 77% against clinical malaria at 12 months of follow-up in a Phase II trial. R21 also induces high titre antibody responses to CSP and after a booster dose, antibody levels returned to a comparable level to the primary response [5].

One of the secondary endpoints for the RTS,S/AS01 clinical trials was the measurement of IgG antibody concentrations to the NANP central repeat region of CSP in the vaccinated participants as a measure of the vaccine’s ability to induce protective immunity [7]. Although the correlates of protection against malaria have not been unequivocally defined, anti-CSP antibodies are associated with RTS,S/AS01 vaccine efficacy [8, 9]. Generally, humoral responses quantification has played a key role in the evaluations and subsequent licensure of several vaccines [10]. ELISA has been used extensively as the gold standard assay for determining the vaccines’ induced antibody levels against infectious diseases. Further, for R21, the highest anti-CSP antibody concentrations measured using a validated ELISA assay correlated with a significant reduction in the risk of clinical malaria [5]. Nonetheless, there is no international standardisation on the ELISA assays used for assessing the anti-CSP-based malaria vaccine immunogenicity and this has hindered direct comparisons of the immunogenicity of leading vaccine candidates.

Following the licensure of RTS,S/AS01 malaria vaccine, the assays used for measuring the antibody levels induced by the subsequent pre-erythrocytic vaccines ought to be comparable to the assays that were pivotal for RTS,S/AS01 licensure during the clinical trials. Moreover, assays standardisation and/or cross-validation allows for the expression of results in international units or equivalent units thus enabling comparisons of the immune responses and the associated clinical effects across vaccines sharing similar antigens [10].

Here, the correlations of the RTS,S/AS01 induced anti-CSP IgG antibody levels from the same participants were established using three different and independently developed ELISA protocols. The ‘Oxford’ ELISA protocol described in this study has been used for serological evaluations of the R21 malaria vaccine during Phase I and II clinical trials [11]. The ‘Kilifi’ protocol has recently been used for retrospective evaluation of the kinetics of RTS,S/AS01 induced humoral responses [12]. Previously, the anti-CSP IgG antibody levels from the RTS,S/AS01 phase IIb and III clinical trials, and others were measured centrally by the Centre for Vaccinology (CEVAC) laboratory, at the University of Ghent, Belgium, using a specific standardised ELISA protocol (hereby referred to as ‘Ghent’ ELISA protocol) [13].

## Methods

### Study design

The current study used 196 plasma samples randomly selected from 447 samples collected from children aged 5–17 months who participated in the randomised, controlled, double-blind RTS,S/AS01 Phase IIb clinical trial in 2007 in Kilifi-Kenya sponsored by GlaxoSmithKline (NCT00380393) [13]. The participants received 3 monthly RTS,S/AS01 vaccine doses. The 196 samples were from 70 children and corresponded to 3, 6.5, 14 and 21 months after receiving the third (final) dose of RTS,S/AS01 vaccine. These time points represent a range of high to low antibody responses as RTS,S/AS01 induced anti-CSP IgG antibodies rapidly peak at month three after the third dose vaccination and wane relatively quickly after month 6.5 after the third dose vaccination [14]. These plasma samples have been stored at -80°C, until the quantification of the anti-CSP IgG antibodies using the ‘Kilifi’ and ‘Oxford’ ELISA protocols.

### Ethical clearance

The main study (i.e. phase IIb clinical trial in 2007) had obtained ethical approval from the Kenya Medical Research Institute National Ethical Committee, the Western Institutional Review Board in Seattle, the Central Oxford Research Ethics Committee and the London School of Hygiene and Tropical medicine Ethics Committee. The parents/guardians provided informed consent for storage and/or future scientific usage of the samples. The current study received ethical clearance from the Scientific and Ethics Review Unit-Kenya Medical Research Institute (SERU 2887). The archived sample information was fully anonymised.

### ‘Kilifi’ ELISA protocol

#### Determining the levels of anti-CSP IgG antibodies

As previously described [12], the RTS,S/AS01 induced anti-CSP IgG antibodies were measured using a standardised ELISA at Kenya Medical Research Institute (KEMRI)-Wellcome Trust Kilifi. Briefly, ninety-six-well plates (Immuno4 HBX- Thermo Scientific^™^) with high absorbance were coated with 100μL per well of 1μg/mL *P*. *falciparum* CSP, (NANP)_9_ repeat region (Biomatik^™^) in carbonate-bicarbonate coating buffer (Sigma Aldrich^™^) pH 9.4 and incubated at 4°C overnight. The plates were washed thrice with 0.05% Tween 20 in phosphate-buffered saline pH 7.2 (PBS-T) using an ELx405 automated machine washer. Then blocking was done for 3 hours for IgG at room temperature by adding 200μL per well of 1% dried skimmed milk powder in PBS-T.

After 3 washes, 100μL per well of diluted plasma samples was added to the duplicate wells at a final dilution of 1:4000 for IgG in PBS-T and incubated at 4°C overnight. On the next day, the plates were washed six times with PBS-T, and pat dried. 100μL per well of horseradish peroxidase-conjugated rabbit anti-human IgG (DAKO^™^) was then added following 1/5000 dilution in PBS-T. The plates were incubated for 3 hours at room temperature. 100μL per well of OPD (Sigma Aldrich^™^) substrate solution was added and left at room temperature in the dark for 15 minutes. The reaction was stopped by adding 25μL per well of 2 molar sulphuric acid (2M H_2_SO_4)_. The optical densities (ODs) were then read at an absorbance of 492nm using a Gen5^™^ microplate reader.

#### Determining the arbitrary anti-CSP IgG antibodies concentrations for the ‘Kilifi’ ELISA protocol

To ensure the accuracy of the results, human plasma samples were tested in duplicate. Plasma samples that exhibited ODs variability >20% were re-tested. An internal positive control from a pooled plasma from the RTS,S vaccinated children was also included on each plate. Besides, malaria-naïve negative controls from Sweden and the United Kingdom were included.

Pooled plasma from 10 participants with the highest antibody titres at three months after the third dose of vaccine was used to establish the standard curves. A two-fold dilution was used for generating the standard curves, starting with 1:2000. The top standard arbitrary concentration was 100. Subsequently, by subtraction of the blank wells’ average ODs to correct for the background reactivity and multiplication with the dilution factor, the arbitrary antibody concentrations for each plate were determined independently using Gene5^™^ analytical software determined in ELISA Units/milliliter (EU/mL).

### ‘Oxford’ ELISA protocol

#### Determining the levels of anti-CSP IgG antibodies

Plasma samples were tested for the presence of RTS,S/AS01 induced anti-CSP antibodies using a standardised ELISA protocol as described elsewhere [11]. Briefly, the plates (Fisher^™^) were coated with 50μLper well of 0.2μg/mL NANP6C antigen in carbonate-bicarbonate buffer (pH 9.6) and incubated overnight at room temperature. On the following day, the coating solution was flicked off and the plates were washed 6 times in 0.05% PBS-T using an ELx405 automated machine washer and pat dried. Blocking was then done with 100μL per well of casein blocking buffer (Thermo Scientific^™^) for 1 hour 30 minutes. The blocker was then flicked off and pat-dried. 50μL of the diluted samples were added at a dilution of 1:4000 in triplicate and the blank control (Casein). Subsequently, 100μL per well of the standard was added starting from the highest concentration, and the internal control was added lastly. The plates were then incubated at room temperature for 2 hours. This was followed by 6 times washing with PBS-T and tap drying. 50μL per well of the detecting antibody (Sigma^™^ goat anti-human IgG Alk Phos A1387) diluted at 1:1,000 in casein was added and incubated at room temperature for 1 hour. The plates were then washed 6 times using PBS-T.

The development solution was made by dissolving the 4-Nitrophenyl Phosphate 20mg Tablets (Sigma^™^) in 1x diethanolamine buffer (Pierce^™^). 100μL of development buffer was added to each well followed by OD reading using Gen5 software at 405nm after 8 minutes of incubation in the dark.

#### Determining the arbitrary anti-CSP IgG antibodies concentrations for the ‘Oxford’ protocol

For accuracy, the plasma samples were tested in triplicate with the inclusion of an internal control in each plate. Triplicate OD values for each sample >20% were re-tested. The standard curves for the Oxford assay were made from pooling a total of sera from 37 volunteers containing high levels of anti-CSP antibodies induced from 3 doses of RTS,S/AS01B from a study titled VAC55, NCT01883609 previously run at the Jenner Institute [15]. A two-fold dilution was used for the standard curves, starting with 1:100. The top standard was given an arbitrary concentration of 20. Subsequently, by subtracting the blank wells’ average ODs to correct for the background reactivity and multiplying with the dilution factor, the arbitrary antibody concentrations for each plate were then determined independently using Microsoft Excel in EU/mL.

### ‘Ghent’ ELISA protocol

The RTS,S/AS01 clinical trial partnership used a standardised and validated ELISA protocol established by the CEVAC in Ghent University, Belgium to determine the anti-CSP IgG antibody levels during the RTS,S/AS01 phase IIb trial in 2007 and 2008 [13]. We conducted a secondary analysis of these data as a potential reference assay to compare with the Oxford and Kilifi ELISA protocols. Similar to Oxford and Kilifi assays, the plates were adsorbed with recombinant antigen R32LR which contains [NVDP (NANP) 15]2LR [16]. The antibody titres were determined in EU/mL.

### Statistical analysis

First, the intraclass correlation coefficient (ICC) was computed for each ELISA protocol to assess overall variation explained by clustering due to repeated anti-CSP IgG antibodies measurements within a child. The ICC measures the relatedness of the measurements within a child and ranges from 0 (measurements within a child are as heterogenous as measurements between children) to 1 (measurements within a child are identical). As all three ICC values were not more than trivial (i.e., <0.05: ICC = 0 for both Kilifi and Ghent, ICC = 0.04 for Oxford), methods for multilevel data were not used.

Second, the ELISA values from each assay were summarised using the median and interquartile range (IQR). Before subsequent correlative analyses, data were log-transformed to normality as they were highly skewed to the right and assessed for outliers. Third, scatterplots and Pearson correlation coefficient between the assays were then determined using the log-transformed values before and after the removal of the potential outliers. Finally, to be able to convert the arbitrary ELISA units into equivalent units for sample *i* we fitted, for each pair of the assays, a simple deming regression model [17]. Deming regression uses paired measurements, (*x*_*i*_, *y*_*i*_), measured with errors *δ*_*i*_ and *ε*_*i*_, where:

$$\begin{array}{l} x_{i}=X_{i}+\delta_{i},\;\delta_{i}\sim N\left(0,\;\tau^{2}\right)\\ y_{i}=Y_{i}+\varepsilon_{i},\;\varepsilon_{i}\sim N\left(0,\;\sigma^{2}\right) \end{array}$$

to estimate the unknown intercept, *β*_0_, and slope, *β*_1_, from the data using the equation

$${\hat{Y}}_{i}=\beta_{0}+\beta_{1}{\hat{X}}_{i}.$$

${\hat{Y}}_{i}$ is the estimate of the “true” (or expected) sample *i* output ELISA assay that we want to find the equivalent of (*Y*_*i*_, say Kilifi assay) and ${\hat{X}}_{i}$ is the estimate of the “true” assay where we already have values for sample *i* (*X*_*i*_, say Ghent assay).

To check the predictive power of the models, the coefficient of determination, graphical diagnostics (such as residual plots, Q-Q plots, and leverage- S1–S3 Figs) and formal tests were observed [18]. For outlier/influential points, Cook’s distance was used. More specifically, samples/observations with Cook’s distance greater than 3 times the standard deviation were considered influential. Outlying observations were excluded and the analysis was repeated.

Furthermore, the conclusion was identical using quantile-based flooring and capping of outliers. To assess agreement between each pair of assays, the limits of agreement method of Bland and Altman was used [19]. As the units were arbitrary, each assay was first standardised by subtracting its mean and dividing by standard deviation before applying the Bland and Altman method.

#### Summary of model fit evaluation

Potential influential observations filtered out for the model predicting Oxford ELISA units using Kilifi protocol were 13 (Model A), for the model predicting Oxford samples using Ghent samples were 6 (Model B), and for the model predicting Ghent samples using Kilifi samples were 11 (Model C). The near-zero residuals and high coefficients of determination (Model A = 0.87; Model B = 0.89; and Model C = 0.94) corroborated this. Statistical analyses were performed using R version 4.1.0 [20], except for the calculation of the analytical ranges which was done using Belysa^®^ immunoassay curve fitting software. All tests were performed at 5% significance level.

## Results

### Comparisons of the measurements of RTS,S/AS01 induced anti-CSP IgG antibodies among the three ELISA protocols

#### Assays analytical characteristics

To facilitate accurate interpretation of the ELISA units among the three protocols, the analytical ranges for assays was determined using Belysa^®^ immunoassay curve fitting software. Specifically, the lower limits of quantification (LLOQ), the upper limits of quantification (ULOQ), and clinical cut-offs for the assays were established. The ‘Oxford’ protocol exhibited the lowest clinical cut-offs, LLOQ, and ULOQ (Table 1).

**Table 1:** The top standard arbitrary concentration values, clinical cut-offs, and the lower and upper limits of quantification for the ‘Oxford’, ‘Kilifi’, and ‘Ghent’ ELISA assays. All results are shown in EU/mL.

ELISA assays analytical ranges	‘Oxford’-Jenner Institute ELISA Assay (EU/mL)	KEMRI-‘Kilifi’ ELISA Assay (EU/mL)	CEVAC-‘Ghent’ GSK ELISA Assay (EU/mL)
Top standard arbitrary concentration	20	100	109
Cut-offs (clinical positivity)	≥0.15	≥0.39	≥0.50
The lower limit of quantification (LLOQ)	0.04	0.20	0.30
The upper limit of quantification (ULOQ)	19.20	95.20	190.00

**Fig 1:**
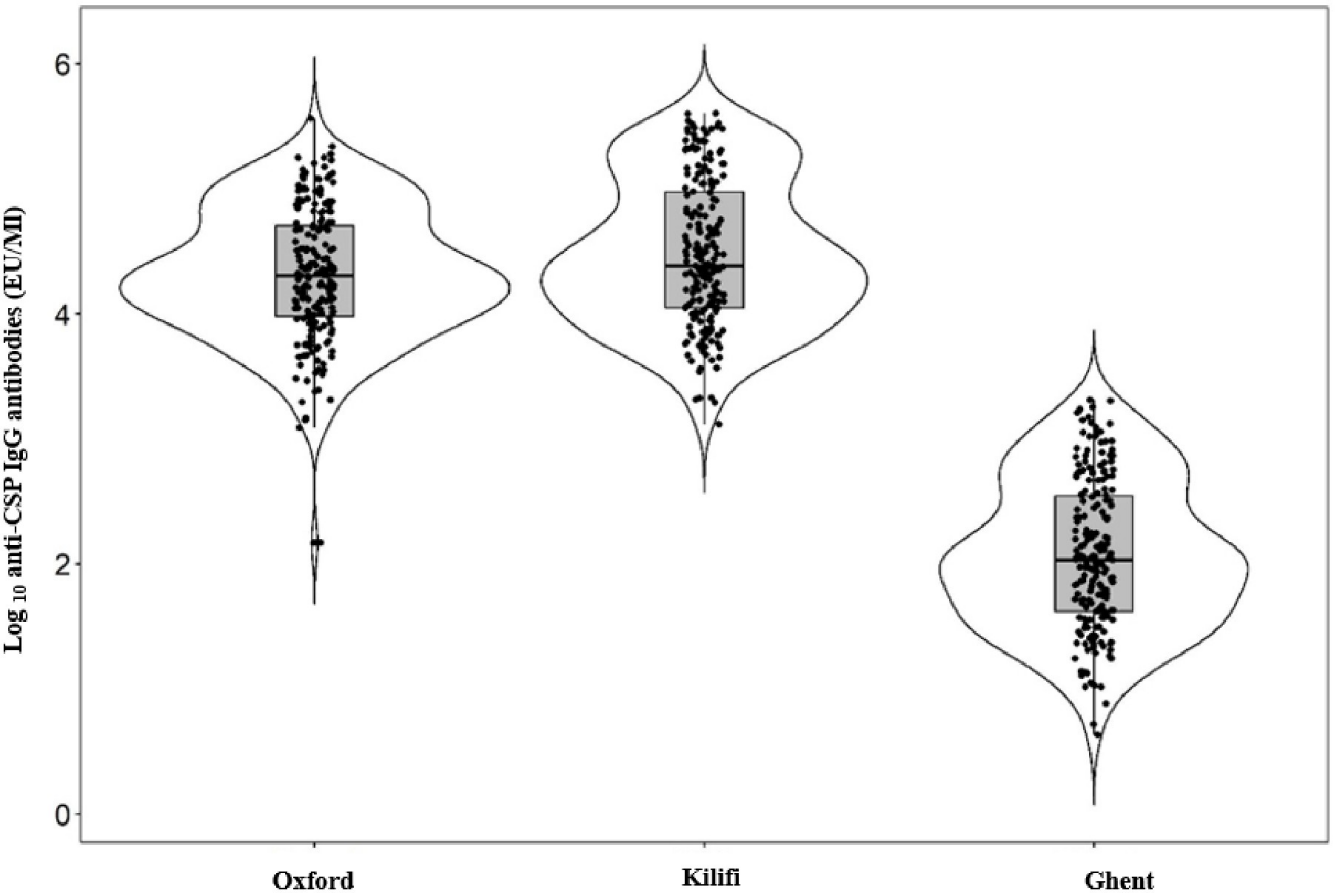
Violin plots showing the log_10_ EU/mL individual distributions and comparisons between the ‘Oxford’, ‘Kilifi’, and ‘Ghent’ ELISA protocols, the middle line represents the medians. The upper and lower whiskers represent the highest and lowest values within 1.5 interquartile ranges, with outliers shown as extreme values, n = 196 for each of the protocols.

The medians (IQR) for the three assays were as follows: ‘Oxford’: 3915 EU/mL (IQR 1871–10117), ‘Kilifi’: 4784 EU/mL (IQR 2225–18873), and ‘Ghent’: 105 EU/mL (IQR 41–328). As reported elsewhere, the baseline (Month 0) anti-CSP measurements were in concordance (close to zero) for the three ELISA assays [13]. Expectedly, the differences in measurements of RTS,S/AS01 induced anti-CSP antibodies among the three protocols were statistically significant (p<0.0001, Mann-Whitney U-test). The Kilifi and Oxford ELISA protocols exhibited relatively higher ELISA Unit values compared to the Ghent protocol (Fig 1).

### Correlations of the RTS,S/AS01 induced anti-CSP IgG antibodies between the three ELISA protocols

**Fig 2:**
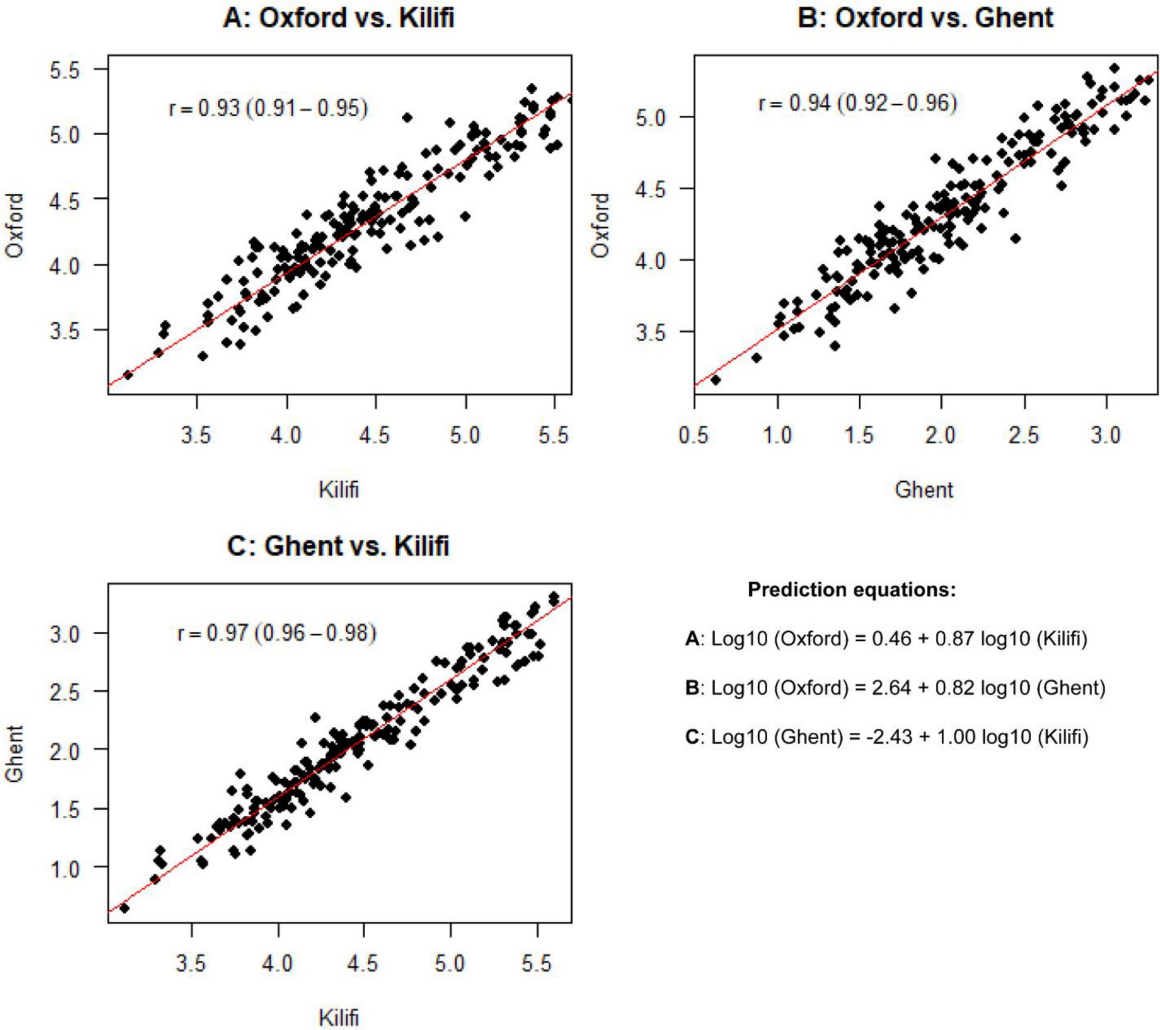
Scatterplots showing strong positive correlations of the anti-CSP IgG antibodies measurements expressed in log_10_ EU/mL among the three ELISA assays. **A**: ‘Kilifi’ and ‘Oxford’, **B**: ‘Oxford’ and ‘Ghent’, and **C**: ‘Ghent’ and ‘Kilifi’ ELISA protocols (n = 196 for all protocols). (bottom right) presents the prediction equations relating to the three assays for the conversion of the arbitrary ELISA units (EU/mL) into inter-assay equivalent units estimated using deming regression model.

The correlations between the three ELISA assays were all strong and significant (Fig 2), they varied from a low of r = 0.93 (95% CI 0.91–0.95) between ‘Oxford’ and ‘Kilifi’ protocols, and a high of r = 0.97 (95% CI 0.96–0.98) between ‘Ghent’ and ‘Kilifi’ protocols. S4 Fig. presents these correlations before excluding the outliers. Surprisingly, from the derived conversion equations, the ‘Oxford’ assays outputs were around 2log_10_ steps above those of the ‘Ghent reference’ assay; highlighting the importance of these equivalent units or international standards for effective comparisons of vaccine immune responses and/or the associated clinical effects.

### Assessment of agreement among the three ELISA protocols

**Fig 3:**
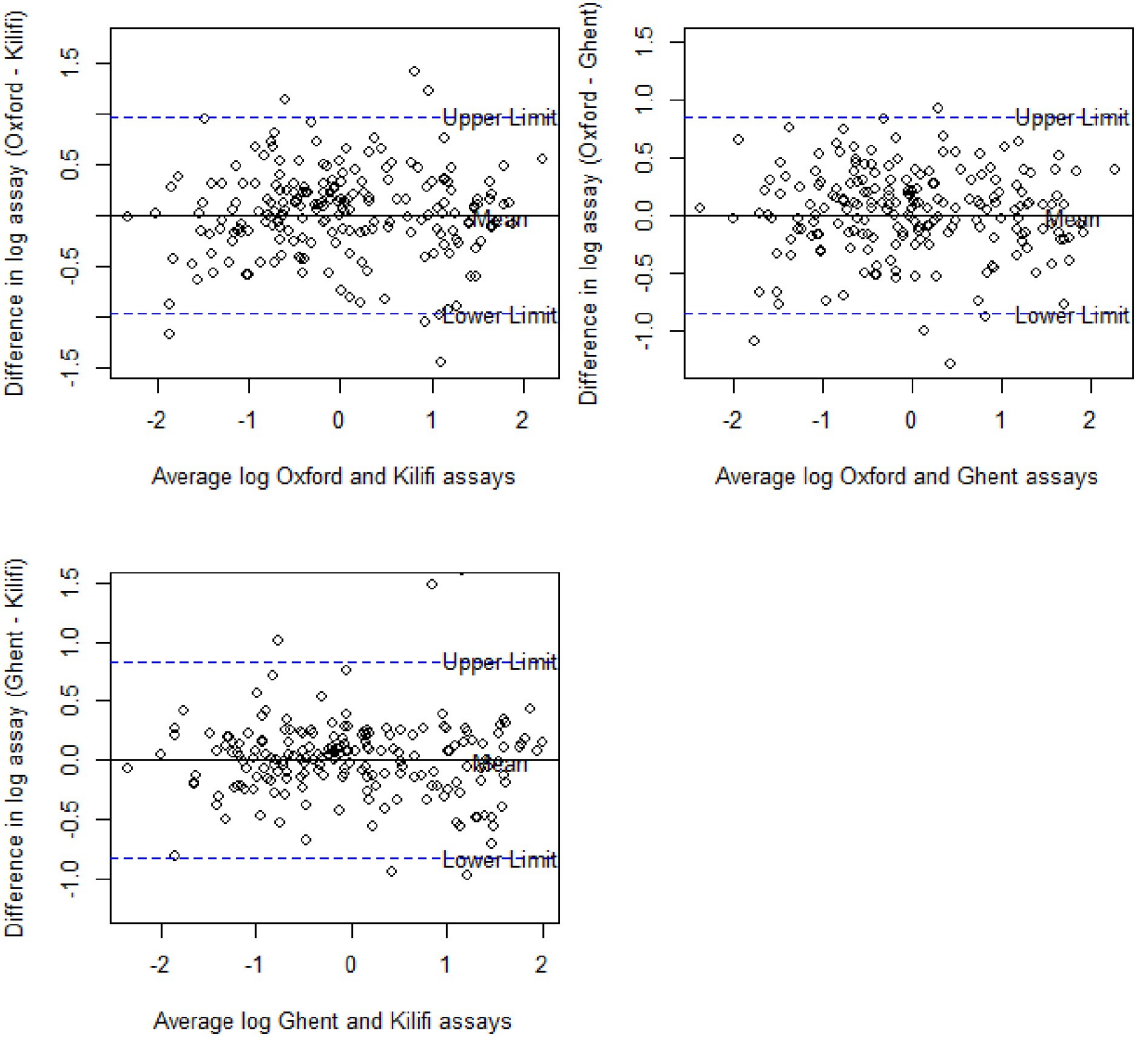
Bland-Altman plots for each pair of the assays. The solid horizontal black line corresponds to the mean difference while the dotted horizontal blue lines correspond to the 95% limits of agreement.

The Bland-Altman plots for assays on log-transformed data show that the assays are generally in agreement. The mean differences were all zero, with limits of agreement from -0.96 to 0.96 for Oxford vs Kilifi, from -0.84 to 0.84 for Oxford vs Ghent, and from -0.83 to 0.83 for Ghent vs Kilifi (Fig 3).

## Discussion

Several malaria vaccine candidates targeting different stages of the malaria parasite have been developed reporting varying degrees of success upon evaluations [21]. RTS,S/AS01 is the only malaria vaccine that has been recommended for use in the expanded programme for immunisation in children at risk of moderate to high *P*. *falciparum* malaria infection [2]. Being the first licensed malaria vaccine, RTS,S/AS01 will serve as a key pathfinder for other malaria vaccines. The characterisation of the future malaria-vaccine immunological responses, assays standardisation, and the assessment of the potency of the vaccine are important issues during clinical development and evaluation [10, 22].

Antibodies against the NANP central repeat region of CSP have consistently been used as a measure of immunogenicity for the RTS,S/AS01 malaria vaccine as this is the primary target of the vaccine-induced humoral response [7, 23]. Although the definitive immune correlates of the RTS,S/AS01 protection has not been established yet, the vaccine-induced anti-CSP IgG antibodies against the NANP region of CSP have been associated with the vaccine efficacy from clinical trials with malaria episodes endpoint [7, 24, 25]. The RTS,S/AS01 phase III clinical trials reported a relatively low vaccine efficacy of 46% over the first 18 months following the 3-dose primary immunisation which wanes over time as the antibody response wanes [4, 9, 26]. Future vaccines with comparatively higher immunogenicity coupled with better efficacy compared with RTS,S/AS01 will be required to reduce the worsening malaria morbidity and mortality trends [6].

Currently, there is no agreed international standard for the standardisation of the assays used for the assessment of RTS,S/AS01 immunogenicity, and/or other CSP-based vaccines. The characterisation of novel malaria vaccine-induced immune responses to the NANP region of CSP similar to that of the licensed vaccine (RTS,S/AS01) can be carried out through measurements of antibody peak concentrations and the kinetics of waning responses via assays such as ELISA. The specific assay used to measure these antibodies should be either commercially available with an international standard or comparable to the reference assay that was used in the initial clinical trials and was key for the subsequent licensing of RTS,S/AS01. The ‘Oxford’ ELISA protocol described in this study has already been used for the assessment of the immunogenicity of the R21/MM vaccine and evaluation of its associated efficacy [5, 11]. Furthermore, clinical trial results generated using assays that are not comparable to those used for the licensed vaccines should not be used to make specific claims regarding differences in immunogenicities and/or efficacies [10, 22].

In this study, we report high correlation coefficients of RTS,S/AS01 induced anti-CSP IgG antibodies measured using three independently developed ELISA assays with different characteristics. This highlights the reliability of the antibody concentrations obtained using any of the protocols. Thus, they can be used comparably for the measurements of the vaccines induced anti-CSP antibodies with a coefficient of correlations of up to 97%. Moreover, these correlations and agreement demonstrate the comparability of the ‘Kilifi’ and ‘Oxford’ ELISA assays to the potential reference ‘Ghent’ ELISA assay.

The linearity established between the antibody measurements suggests that ELISA serological data from the second generation of anti-circumsporozoite vaccines using either of the assays can precisely be compared to that of RTS,S/AS01 by converting ELISA units into equivalent units. The linear relationships between the ELISA values across different protocols show their ability to test anti-CSP antibody levels, which are directly proportional to the antibody concentration in the samples [27]. By making use of the established equations, the results from either of the ELISA assay can easily be converted into more comparable outputs.

However, caution should be applied when comparing the results of trials in different populations. The immunogenicity of RTS,S/AS01 has varied by site in Phase III studies and the determinants of immunogenicity are incompletely understood [4]. Furthermore, other factors like malaria annual seasonality might greatly influence vaccine-induced immune responses [28]. In this view, administration of RTS,S/AS01 before the peak of malarial seasons in areas of highly seasonal malaria transmission resulted in the reduction of malarial cases by up to 75% [29].

The deming regression model used to analyse these data correctly accounts for random measurement errors for both the predicted and predictor variables (in this case, the predicted and predictor ELISA protocols) [17, 30]. However, it does not take into account the fact that a child could have measurements taken at more than one time point. The basic statistical prerequisite for the appropriate application of multilevel modeling includes clustered data with a positive ICC. A positive ICC violates the independent observations assumption resulting in downwardly biased standard error estimates, overly large test statistics, and inflated type 1 error rates [31]. In our analyses, independence of observations was assumed as the ICC value at the child level was nought for all ELISA protocols except for Oxford but which was also not more than trivial (i.e., <0.05). Otherwise, a method extending the model of deming to accommodate multilevel data would be more appealing.

In conclusion, the high correlations and agreement of the three ELISA assays in this study and the establishment of the ELISA unit conversion equations allow for accurate comparisons of CS antibody levels when measured by different assays. In the absence of international standardisation, the concept of ELISA unit conversion equations is the only alternative for the subsequent evaluation across different studies.

## Supporting information

S1 Fig Diagnostic plots for Model A. (DOCX)

S2 Fig Diagnostic plots for Model B. (DOCX)

S3 Fig Diagnostic plots for Model C. (DOCX)

S4 Fig Scatterplots showing strong positive correlations of the log_10_ arbitrary ELISA units between the three assays before excluding the influential outliers. (DOCX)

S1 File Dataset for the three ELISA protocols. (XLSX)

## Abbreviations

AEUs: Arbitrary Elisa Units
CEVAC: Centre for Vaccinology (Ghent)
CSP: Circumsporozoite protein
ELISA: Enzyme-Linked ImmunoSorbent Assay
EU/mL: ELISA Units/milliliter
HRP: Horseradish peroxidase
IgG: Immunoglobulin G
IQR: interquartile ranges
KEMRI: Kenya Medical Research Institute
LLOQ: Lower limits of quantification
M: Month
NANP: Peptide representing the central repeat region of CSP
OD: Optical density
OPD: o-Phenylenediamine
PBS: Phosphate buffered saline
ULOQ: upper limits of quantification
