# Supplementary figures and images for: Correlations between three ELISA protocols measurements of RTS,S/AS01-induced anti-CSP IgG antibodies
Source: PLoS One. 2023 May 23;18(5):e0286117. doi: 10.1371/journal.pone.0286117 (PMC10204981; doi:10.1371/journal.pone.0286117)

**S1 fig.**
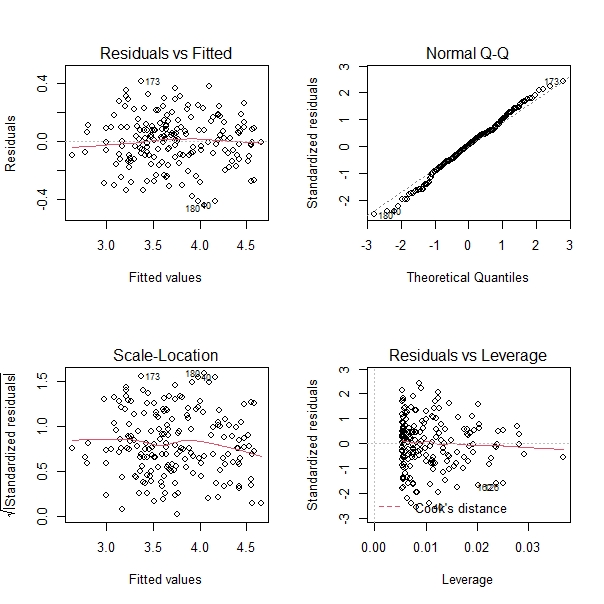

Supplement: S1 Fig — (DOCX) [file pone.0286117.s001.docx]

**S2 fig.**
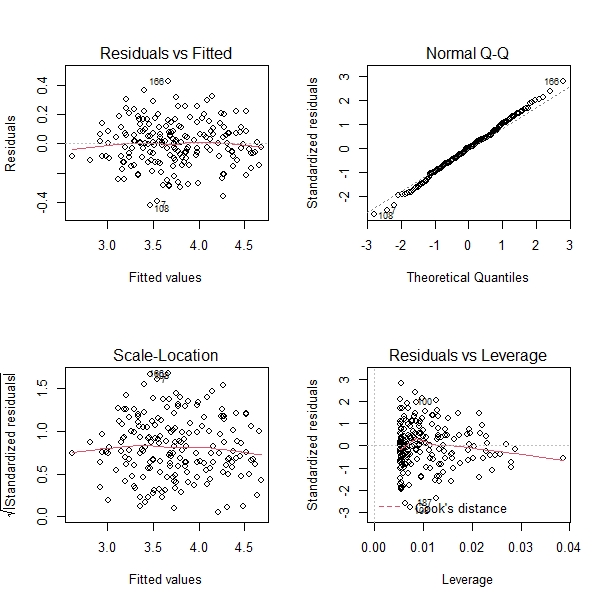

Supplement: S2 Fig — (DOCX) [file pone.0286117.s002.docx]

**S3 fig.**
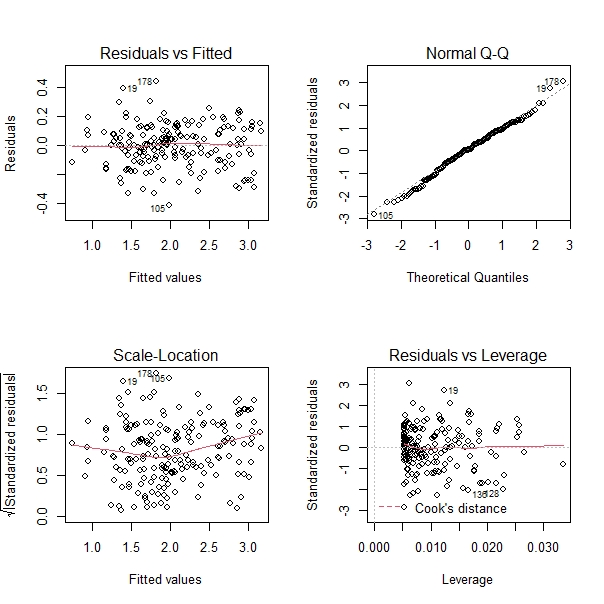

Supplement: S3 Fig — (DOCX) [file pone.0286117.s003.docx]

**S4 fig.**


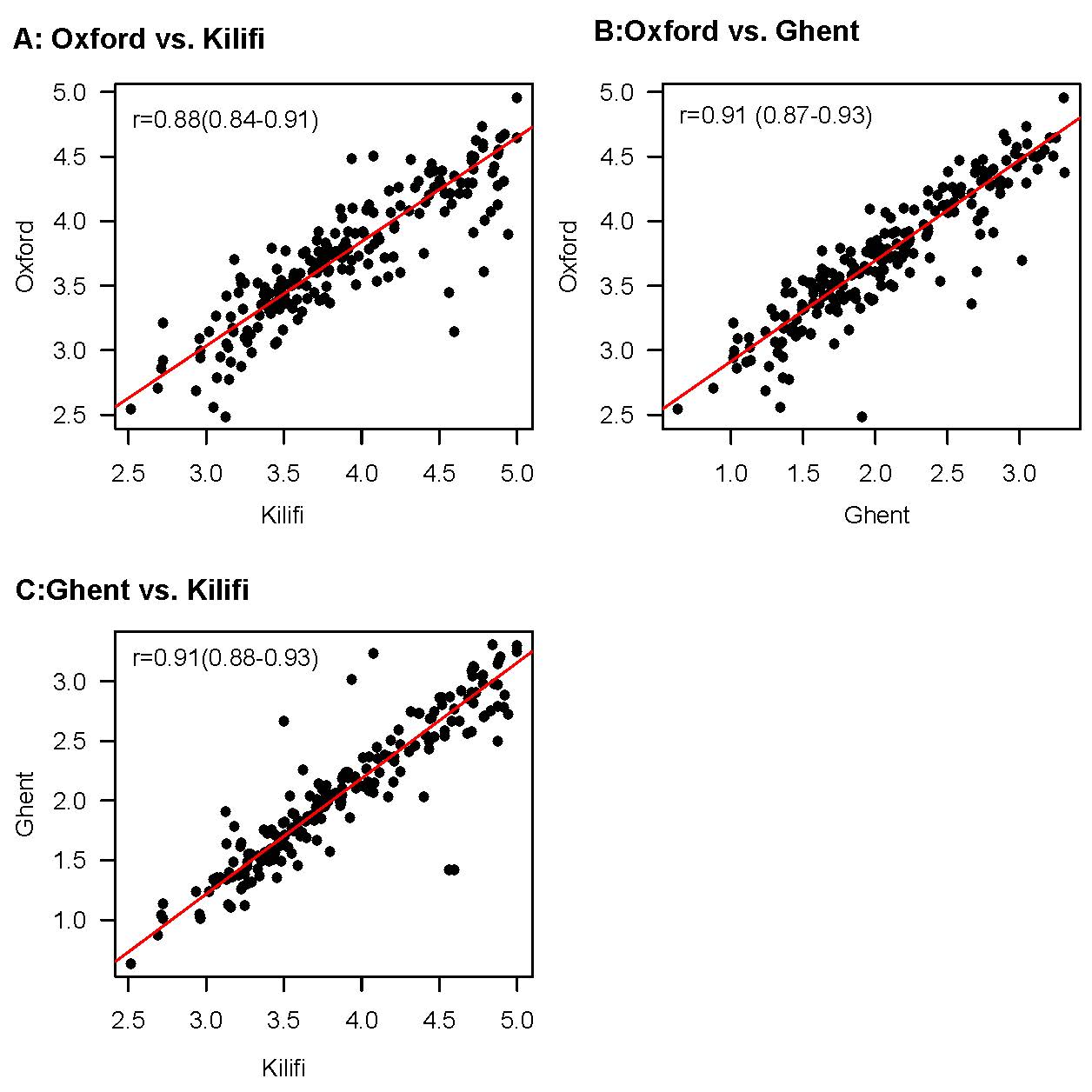

Supplement: S4 Fig — (DOCX) [file pone.0286117.s004.docx]
